# The Mediation Effect of Coping Style on the Relations between Personality and Life Satisfaction in Chinese Adolescents

**DOI:** 10.3389/fpsyg.2017.01076

**Published:** 2017-06-29

**Authors:** Le Xu, Ru-De Liu, Yi Ding, Xiaohong Mou, Jia Wang, Ying Liu

**Affiliations:** ^1^Beijing Key Laboratory of Applied Experimental Psychology, Faculty of Psychology, Beijing Normal UniversityBeijing, China; ^2^Graduate School of Education, Fordham University, New YorkNY, United States; ^3^Beijing PolytechnicBeijing, China

**Keywords:** the Big Five, personality traits, coping style, life satisfaction, Chinese adolescents

## Abstract

Previous findings showed the associations between each of the Big Five personality trait and adolescents’ life satisfaction were different. Some traits (extraversion and neuroticism) correlated with adolescents’ life satisfaction, while other traits did not have the same associations with adolescents’ life satisfaction. In order to explain why the Big Five traits differed in their associations with adolescents’ life satisfaction, the present study verified the relations between each of the Big Five personality traits and life satisfaction, and demonstrated the mediating effects of coping style on the relations between these personality traits and life satisfaction in a sample of 2,357 Chinese adolescents. The results demonstrated that four of the Big Five personality traits (extraversion, agreeableness, conscientiousness, and neuroticism) had significant associations with life satisfaction. Further, coping style partially mediated the relations between these four traits and life satisfaction, whereas coping style fully mediated the relation between openness to new experience and life satisfaction. The results implied a plausible explanation for why the Big Five traits differed in their associations with life satisfaction found among the previous literature: that there might be some partial or full mediation variables (such as coping style in this study) left unexamined. Theoretical and practical implications of this study on further research and educational practice are discussed.

## Introduction

Subjective well-being has been a focus in positive psychology (Seligman and Csikszentmihalyi, 2000). As an important cognitive component and indicator of subjective well-being (Diener et al., 1999), life satisfaction has recently gained increasing research attention. Life satisfaction refers to an individual’s evaluation of the quality of all aspects of life, including study, work, experiences, relationships with others, living environment, and so on, based on self-defined standards (Diener and Diener, 1995). Life satisfaction is a subjective cognitive judgment, and the level reported by an individual may change with some life events or be unstable during the entire lifespan (Deniz, 2006). During the past 20 years, the focus of studies on life satisfaction has shifted from exploring its measurement to analyzing its influential factors. An individual’s life satisfaction is influenced by subjective variables, such as personality (James et al., 2012), self-esteem (Joshanloo and Afshari, 2011), self-concept (McCullough et al., 2000), and coping style (Sun and Tao, 2005), and by contextual variables, such as life events (McKnight et al., 2002), relationships with others (Man, 1991; Nickerson and Nagle, 2004; Lowenstein et al., 2007), and social culture (Liu, 2006). Compared to contextual variables, subjective variables are more stable and internal (Eysenck, 1983) and have attracted increased attention of researchers. Numerous studies have explored the relation between personality and life satisfaction, discussing that people with different traits have shown different levels of satisfaction for their work, relationships, and so on. However, the Big Five traits showed different associations with life satisfaction, and there have been inconsistent conclusions on these associations. This study aimed to explain these differentiated associations through exploring the mediating mechanisms underlying personality traits and life satisfaction.

### Personality and Life Satisfaction

Most of the studies about the relations between personality traits and life satisfaction were based on the model of the Big Five. From the viewpoint of trait theory, the Five Factor Model (FFM) represents a classical structure of personality (John et al., 2008). The Big Five personality traits originated from lexical tradition (Goldberg, 1981) and include extraversion, agreeableness, openness to new experiences, conscientiousness, and neuroticism (McCrae and John, 1992; McCrae and Costa, 2008). *Extraversion* refers to the quantity and intensity of a person’s social communication, and the degree of energy and happiness a person feels during communication. *Agreeableness* reflects attitudes toward other people. *Openness to new experiences* explores whether a person has creativity and imagination, and is likely to try new things. *Conscientiousness* refers to self-control regarding conflicts or actions. *Neuroticism* reflects a person’s emotional instability and inability to adapt to the environment (McCrae and John, 1992). The FFM has been confirmed in different cultures (Rolland, 2002; Terracciano et al., 2005; Schmitt et al., 2007).

Many trait studies and meta-analyses have found that some personality traits such as extraversion and neuroticism were highly correlated with life satisfaction (DeNeve and Cooper, 1998; Schimmack et al., 2002; Diener et al., 2003; Suldo et al., 2015), where extraversion positively correlated with life satisfaction, whereas neuroticism negatively correlated with life satisfaction. However, it appears that not all of the five factors have the same association with life satisfaction for adolescents; some factors strongly correlate with life satisfaction, but others do not (Joshanloo and Afshari, 2011). These findings have been shown in a number of studies. For example, Garcia (2011) concluded that only extraversion and neuroticism had strong associations with adolescents’ life satisfaction. Extraversion had a positive correlation with adolescents’ life satisfaction, but neuroticism had a negative correlation with adolescents’ life satisfaction, which was consistently verified by previous studies (e.g., Fogle et al., 2002; McKnight et al., 2002; Rigby and Huebner, 2005; Ho et al., 2008; Wang, 2013). However, agreeableness, openness to new experiences, and conscientiousness did not have significant association with adolescents’ life satisfaction (Garcia, 2011). Furthermore, Wang (2013) reported that neuroticism as well as openness to new experiences was negatively correlated with adolescents’ life satisfaction, while extraversion as well as conscientiousness was positively correlated with adolescents’ life satisfaction. In short, the Big Five traits differed in their associations with life satisfaction (e.g., Lounsbury et al., 2005; Suldo et al., 2015).

In order to explain why the Big Five traits had different association with life satisfaction, some previous research has explored the mediating or moderating mechanisms that link personality traits and life satisfaction. For example, Steel et al. (2008) studied the moderator roles of age, gender, self-report versus other personality reports and type of sample between personality traits and life satisfaction. Joshanloo and Afshari (2011) concerned about the mediation effects of self-esteem between personality traits and life satisfaction, while Vater and Schröder-Abé (2015) explored the mediation effect of emotion regulation in this link. Different personality traits predispose individuals to different status of their emotional regulation (Vater and Schröder-Abé, 2015). Individuals with high extraversion might experience more positive affection (Larsen and Ketelaar, 1991) and seldom suppress their emotions (Vater and Schröder-Abé, 2015), whereas individuals with high neuroticism might have more negative affection (Larsen and Ketelaar, 1991) and might activate ineffective emotional regulation strategies (Vater and Schröder-Abé, 2015), which further negatively affect their life satisfaction (Tsukerman, 2013). However, positive affection promotes the use of effective coping style (Folkman and Moskowitz, 2000) and enhances problem-solving in stressful situations (Isen et al., 1987; Congard et al., 2011). It is necessary to explore the relation among personality traits, coping style, and life satisfaction.

Personality is believed to influence the quality of life through the ways people react to stressful situations and cope with challenges (Vollrath, 2001; Wrosch and Scheier, 2003; Vollrath and Landolt, 2005; Aarstad et al., 2008). Personality traits are believed to influence individuals’ interpretation of their environments and also affect individuals’ behavioral choices about how to solve encountered problems (James and Mazerolle, 2002; Wayne et al., 2004). With different personality traits, people have different standards to evaluate the level of stress in each situation. One situation may be perceived as extremely stressful by some people, whereas other people may easily adapt to the same situation (Cüceloglu, 1991; Deniz, 2006).

### Personality and Coping Style

Personality is a dynamic and organized system of characteristics, which uniquely influences individuals’ cognitions, motivations and behaviors in different situations (Ryckman, 2004). Personality is increasingly stable over time and reaches the highest level of stability in individuals’ later life (Roberts and DelVecchio, 2000). Based on the personality trait theory, personality can be regarded as a collection of traits that are highly individualized and relatively stable throughout one’s lifespan, which uniquely influence one’s behaviors or responses to a situation (Suldo et al., 2015). The way people react to stressful situations is referred to as coping style. More specifically, coping style is defined as the strategies a person employs to consciously regulate his physiology, cognition, emotion, and behavior when contending with stressful or difficult situations (Compas et al., 2001; Skinner et al., 2003). Coping style has been classified as problem focused and emotion focused (Folkman et al., 1986), which was expanded later to positive coping (PC) style and negative coping (NC) style, respectively (Chen et al., 2000; Bowleg et al., 2004). PC style involves utilizing positive strategies to deal with problems, such as thinking about different methods to solve problems and seeking social support, whereas NC style involves using negative strategies, such as denial and depression, to escape solving problems. The Big Five personality traits have been found to differently influence individuals’ coping styles (McCrae and Costa, 1986; Gallagher, 1996; Connor-Smith and Flachsbart, 2007; Carver and Connor-Smith, 2010; Baltes et al., 2011; Beauchamp et al., 2011). The personality-coping-outcome theory assumes that when one encounters stressful situations, personality influences one’s coping style differently, which in turn further affects one’s adjustment outcomes (Gallagher, 1996).

Extraversion involves the tendency to actively interact outside oneself, which is described as enthusiastic, assertive, and outgoing (Caspi, 1998). Extraversion has been found to positively relate to PC style (Fickova, 2009; Hambrick and McCord, 2010). Individuals with high extraversion tend to communicate with others, adjust their mood, and believe in their ability to deal with a stressful situation (Caspi, 1998). They are more likely to use problem-focused coping styles (Connor-Smith and Flachsbart, 2007) and PC styles (McCrae and Costa, 1986; Mirnics et al., 2013).

Agreeableness is characterized by friendliness, kindness, helpfulness, likeability, and trust. Individuals with high agreeableness are more likely to have more social networks, which allow them to seek social supports and rely more on PC styles (McCrae and John, 1992; Connor-Smith and Flachsbart, 2007; Baltes et al., 2011). Agreeableness also has been declared to be positively associated with problem-focused coping styles (Baltes et al., 2011) and PC styles (Fickova, 2009; Hambrick and McCord, 2010). Meanwhile, agreeableness has been found to be negatively correlated with NC styles (Connor-Smith and Flachsbart, 2007; Fickova, 2009).

Openness to new experiences has been described as having wide interests, curiosity, and imaginative and artistic tendencies (Caspi, 1998). When coping with difficulties, openness to new experiences leads individuals to restructure cognition for stressful situations or cherish the illusion of reality. It has been reported to be positively associated with PC styles (Fickova, 2009; Hambrick and McCord, 2010).

Conscientiousness is pertinent to a tendency to be organized, intentional, and responsible (Caspi, 1998). Conscientiousness promotes individuals’ adjustment to their coping strategies in a way that allows them to focus their energy on problem solving. Conscientiousness has been negatively correlated with NC styles, such as denial and substance use (Connor-Smith and Flachsbart, 2007; Fickova, 2009) and positively correlated with problem-focused coping styles (Baltes et al., 2011) and PC styles (Hambrick and McCord, 2010).

Neuroticism deals with a tendency to assess situations with negative emotions, such as anxiety, depression, nervousness, fear, and guilt, which influences people to produce negative reactions to escape stressful conditions (Eby et al., 2005). People with low neuroticism are more likely to use problem-focused coping styles, while people with high neuroticism tend to rely on emotion-focused coping styles (Connor-Smith and Flachsbart, 2007). Neuroticism has been positively related to emotion-focused coping styles (Baltes et al., 2011) and NC styles such as withdrawal, denial, and wishful thinking (Connor-Smith and Flachsbart, 2007; Fickova, 2009), and it has been negatively associated with PC styles (Fickova, 2009; Hambrick and McCord, 2010).

### Coping Style and Life Satisfaction

PC styles have been found to be highly correlated with life satisfaction (McCrae and Costa, 1986). Individuals who cope with stress in a positive style will feel more hopeful for the future (Baltas and Baltas, 1996), and have a high level of satisfaction with their quality of life. Saha et al. (2014) reported that a PC style such as seeking social support strategies enhanced adolescents’ satisfaction with friends and with their global lives.

Coping with stress can also influence individuals’ mental health (Cook and Heppner, 1997; Miller et al., 1998) and adjustment outcomes, and then can affect their life satisfaction. Individuals who use coping strategies that focus on problem-solving, namely PC styles, have reported much more positive adjustment outcomes, while individuals who use NC styles (emotion-focused coping) have reported less positive adjustment outcomes (Judge, 1998; Essex et al., 1999; Reichman et al., 2000; Abbeduto et al., 2004; Stoneman and Gavidia-Payne, 2006). The adjustment outcomes correlate with individuals’ satisfaction with their life quality (Burgess et al., 2000; Van De Ven and Engels, 2011).

The most stressful problems for students are related to school or personal life (Ültanır, 1998). Adolescents often encounter increasingly complicated life events: friendships, school, family, and their own psychological challenges. Especially for Chinese adolescents, their adolescence is situated in one of the busiest learning stages during the entire lifespan. In addition to friendship problems and psychological challenges, Chinese adolescents encounter a variety of coursework and academic tasks, and the academic burden can dramatically increase in comparison to the elementary school years (Song et al., 2010; Mou, 2015). Adolescents with good social relationships, academic achievement, and physical health are more likely to have higher levels of life satisfaction (Suldo and Shaffer, 2008). Adolescents who have the ability to cope with stress through healthy lifestyles are more likely to positively evaluate their life satisfaction (Wang and Ding, 2003; Yang et al., 2005; Yue et al., 2006; Saha et al., 2014).

### The Present Study

The indirect evidence cited in the present study about the relations among personality traits, coping style, and life satisfaction have implied that coping style may act as one of the mediation variables between personality traits and life satisfaction (Zhou et al., 2017). From another perspective, the mediation effect of coping style on the relation between personality and quality of life has been confirmed (Burgess et al., 2000; Van De Ven and Engels, 2011), but the examination of the potential mediating link of coping style between personality traits and life satisfaction has not received adequate attention. It seems reasonable to presume that personality traits influence coping style; in turn, coping style influences the evaluation of life experience. One of the purposes of this study was to extend our understanding of the association among personality traits, life satisfaction, and coping style.

Previous studies proved that the Big Five personality traits had differentiated associations with life satisfaction. Some traits such as extraversion and neuroticism were significantly correlated with life satisfaction, while some traits were not significantly correlated with life satisfaction. The differentiated association between the Big Five Traits and life satisfaction might be better explained once the mediating effect of coping style on the relations between personality traits and life satisfaction has been confirmed. It is plausible that coping style fully mediates the relations between some personality traits and life satisfaction, which probably causes the non-significant direct effects between these traits and life satisfaction. The other purpose of this study was to confirm this possible explanation. Here, we simultaneously examined the relations between all personality traits and life satisfaction.

In addition, age (Steel et al., 2008; Wang, 2013) and cultural context were (Shao et al., 2013) related to the link between personality and life satisfaction. Some personality traits, such as openness to new experiences (Suldo et al., 2015; Mou, 2015), were linked with life satisfaction in adolescent’s sample, but they were not correlate with life satisfaction in adults sample (Lounsbury et al., 2005; Joshanloo and Afshari, 2011; Fagley, 2012). Examining how adolescents cope with their stressful life events adds developmental evidence to the existing research. Furthermore, unlike Western countries, the one-child policy in China was in place from 1978 to 2016. Most of Chinese adolescents do not have siblings, which largely affected both Chinese adolescents’ personality development and the association between personality and their life satisfaction (Wang et al., 2002; Shao et al., 2013). This study could advance our understanding on the relation among personality traits, coping style, and life satisfaction in Chinese adolescents.

In summary, the existing research provided preliminary knowledge about the relations among the Big Five personality traits, coping style, and life satisfaction in adolescents. This study aimed to empirically verify that the associations between personality traits and life satisfaction may be mediated by coping styles in Chinese adolescents, and then to utilize the full or partial indirect mediating roles of coping style to explain why the Big Five traits differed in their associations with adolescents’ life satisfaction.

This study examined the following hypotheses based on the previous studies and the rationales above.

H1: Extraversion significantly positively predicts adolescents’ life satisfaction; neuroticism significantly negatively predicts adolescents’ life satisfaction. It is uncertain whether agreeableness, conscientiousness, and openness to new experiences significantly predict adolescents’ life satisfaction.
H2: Coping style plays a mediating role between the Big Five personality traits and adolescents’ life satisfaction.

## Materials and Methods

### Participants

Participants were 2,357 students (average age = 15.6 years, *SD* = 1.32) recruited from eight regular secondary schools in a mid-size city in China; 1,021 were males (43%) and 1,336 were females (57%).

### Ethics Statement

The study was approved by the ethical committee of the School of Psychology at Beijing Normal University. Written informed consents were obtained from the schools, teachers, parents, and all participants prior to initiating the study. All participants were informed that they had the right to withdraw from this study at any time.

### Measures

#### Personality

The Big Five personality traits were measured using the scale (Mou, 2015) that was revised based on the Adolescents’ Five-Factor Personality Questionnaire (Liang et al., 2012). We kept the high factor loading items on low order traits and edited the description of some items in order to help students understand and read items easily. This scale included five dimensions: extraversion (eight items), agreeableness (seven items), openness to new experiences (six items), conscientiousness (nine items), and neuroticism (six items). Each item was responded to on a 5-point Likert-type rating scale ranging from *do not like you at all* (1) to *like you very much* (5) (see Appendix). The coefficient alphas for extraversion, agreeableness, openness to experience, conscientiousness, and neuroticism were 0.86, 0.80, 0.80, 0.85, and 0.80, respectively. The confirmatory factor analysis of this scale was conducted at item level, and the overall fit index of the scale was acceptable: χ^2^ = 12877.72, *df* = 584, χ^2^/*df* = 22.05, *p* < 0.001; RMSEA = 0.06; SRMR = 0.07; CFI = 0.82; GFI = 0.87; NFI = 0.81. The results of the structural equation model (SEM) showed that all factor loadings were between 0.76 and 0.91. There were no cross loadings between factors. The results revealed that the personality scale had acceptable reliability and factorial validity.

#### Coping Style

**FIGURE 1:**
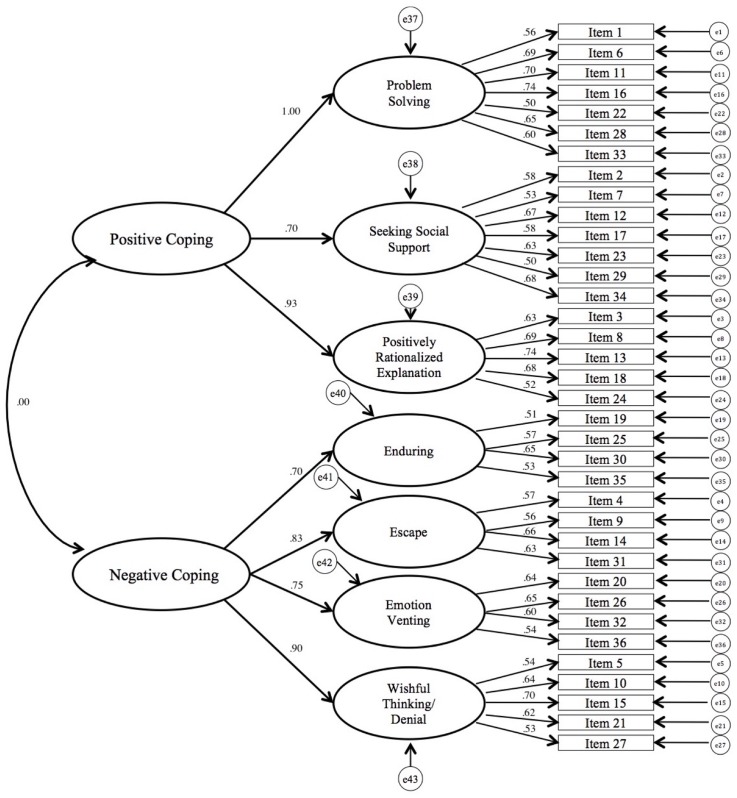
Factor loadings and factor intercorrelations for coping style. All standardized path coefficients are significant with *p* < 0.001.

This study used the Coping Style Scale for Middle School Students (Chen et al., 2000) to assess participants’ coping styles. This questionnaire was developed on the basis of Folkman’s interactive theory, self-regulation theory, and Ways of Coping Questionnaire (WOC; Folkman and Lazarus, 1980). It had 36 items and was rated on a 4-point Likert-type rating scale ranging from *seldom use* (1) to *frequently use* (4) (see Appendix). By adapting the two categories of coping style (Folkman et al., 1986), emotion-focused coping and problem-focused coping, this scale classified coping styles in PC and NC dimensions. Positive coping consisted of three factors: problem solving (seven items), seeking social support (seven items), and positively rationalized explanation (five items), and the coefficient alpha for each was 0.83, 0.78, and 0.78, respectively. NC consisted of four factors: enduring (four items), escape (four items), emotion venting (four items), and wishful thinking/denial (five items), and the coefficient alpha for each was 0.67, 0.68, 0.74, and 0.76, respectively. The coefficient alphas for positive and NC were 0.83 and 0.79, respectively. The coefficient alpha for the full scale was 0.92. Furthermore, the split-half reliability of this scale was 0.88, and its test-retest reliability was 0.89 (Chen et al., 2000). In the current study, the coefficient alphas for problem solving, seeking social support, and positively rationalized explanation were 0.80, 0.76, and 0.73, respectively, and for the PC dimension was 0.88. The coefficient alphas for enduring, escape, emotion venting, and wishful thinking/denial were 0.46, 0.63, 0.69, and 0.74, respectively, and for the NC dimension was 0.81. The confirmatory factor analysis of this scale was conducted at item level, and the overall fit index of the scale was acceptable: χ^2^ = 6755.98, *df* = 586, χ^2^/*df* = 11.53, *p* < 0.001; RMSEA = 0.06; SRMR = 0.08; CFI = 0.88; NFI = 0.80; IFI = 0.88. We specified the SEM of this scale in **Figure 1**, in which the factor loadings and factor intercorrelations of coping style are provided. PC (higher order factor) consists of three lower order factors: problem solving, seeking social support, and positively rationalized explanation, whose factor loadings were between 0.70 and 1.00. There were 19 items related to these three lower order factors, and their factor loadings were between 0.50 and 0.74. NC (higher order factor) consists of four lower order factors: enduring, escape, emotion venting, and wishful thinking/denial, whose factor loadings were between 0.70 and 0.90. There were 17 items related to these four lower order factors, and their factor loadings were between 0.51 and 0.70 (see **Figure 1**). There were no cross loadings between factors. The results revealed that the coping style scale had acceptable reliability and factorial validity.

#### Life Satisfaction

The Multidimensional Students’ Life Satisfaction Scale in the Chinese Version (MSLSS-CV, Tian and Liu, 2005) was used to assess students’ life satisfaction. The MSLSS-CV was translated from the Multidimensional Students’ Life Satisfaction Scale (Huebner and Gilman, 2002), which was a self-report questionnaire. The MSLSS-CV described five domains of life: family, friends, school, living environment, and self. Each of these five factors had five items. Each item was rated on a 4-point Likert-type rating scale ranging from *strongly disagree* (1) to *strongly agree* (4) (see Appendix). We added the rating points of each domain and then divided them by the number of items in this domain to score the overall life satisfaction. A higher score on the MSLSS-CV represented a higher level of life satisfaction. The Cronbach’s α for the MSLSS-CV was 0.90, and the Cronbach’s α for family, friends, school, living environment, and self was 0.87, 0.70, 0.82, 0.71, and 0.72 (*p* < 0.01), respectively. The test-retest reliability for the MSLSS-CV was 0.86. Furthermore, the criterion-related validity for this scale was 0.89, and the corrected item-total correlation was between 0.71 and 0.80 (Tian and Liu, 2005). In this study, the internal consistency coefficient of each factor was between 0.81 and 0.88, and the total internal consistency coefficient was 0.92. The confirmatory factor analysis of this scale was conducted at item level, and the overall fit index of the scale was acceptable: χ^2^ = 3916.98, *df* = 270, χ^2^/*df* = 14.51, *p* < 0.001; RMSEA = 0.06; SRMR = 0.06; CFI = 0.95; GFI = 0.90; NFI = 0.94. The results of the SEM showed that all factor loadings were between 0.68 and 0.87. There are no cross loadings between factors. The results revealed that the life satisfaction scale had acceptable reliability and factorial validity.

### Procedure

Participants completed the three questionnaires individually in their classrooms. They were assured of the confidentiality of their responses, and also were informed that the questionnaires were anonymous and there were no right or wrong answers. Their basic demographic information including gender and age was requested. Trained research assistants instructed participants to answer questionnaires truthfully, supervised participants’ actions, and prepared to help participants who had any questions about the questionnaires in each classroom. The three questionnaires were presented in the Chinese language and took about 30 min to complete.

### Data Analysis

Descriptive statistics and correlations among the main variables were calculated first by SPSS 23.0. Then, to determine whether coping style mediated the relations between the Big Five personality traits and life satisfaction, an SEM was conducted (Joreskog and Sorbom, 1979) by AMOS 17.0, wherein a bootstrap estimation process (with 1000 bootstrap samples) was utilized.

## Results

### The Common Method Bias Examination

To reduce the common method bias, the participants were asked to answer questionnaires anonymously, and some items were designed with reverse scores. However, data for this study were collected by questionnaires that were self-reported by participants. Therefore, in addition to controlling the common method bias via designed procedure that was described above, there was a need to control the common method bias via statistical calculation. First, we examined the common method bias according to the single factor test method of Harman (Podsakoff et al., 2003). Results of this examination indicated that the first un-rotated factor only explained the variance of 18.57%, which was far less than the critical value. Second, we regressed life satisfaction on all the Big Five personality traits in the mediation analysis of coping style.

### Descriptive Statistics and Correlations

General means, standard deviations, and correlations for major variables are shown in **Table 1**. Results suggested that all variables were significantly correlated with each other, except that extraversion did not link with NC.

**Table 1:** Descriptive statistics and correlations among major variables (*N* = 2,357).

Variables	*M*	*SD*	1	2	3	4	5	6	7	8
1. E	3.48	0.75	1							
2. A	4.00	0.59	0.50**	1						
3. O	3.70	0.70	0.52**	0.42**	1					
4. C	3.62	0.66	0.38**	0.57**	0.42**	1				
5. N	2.71	0.86	-0.16**	-0.14**	0.07**	-0.10**	1			
6. PC	3.02	0.47	0.38**	0.42**	0.35**	0.46**	-0.11**	1		
7. NC	2.35	0.49	0.00	-0.07**	0.08**	-0.14**	0.45**	0.09**	1	
8. LS	3.21	0.44	0.45**	0.42**	0.25**	0.42**	-0.34**	0.46**	-0.20**	1

^∗^*p< 0.05; ^∗∗^*p* < 0.01; aaa*p* < 0.001. E = Extraversion, A = Agreeableness, O = Openness to New Experience, C = Conscientiousness, N = Neuroticism, PC = Positive Coping, NC = Negative Coping, LS = Life Satisfaction*.

### Regression Analyses

#### The Associations between the Big Five Personality Traits and Life Satisfaction

To further examine the correlations between the Big Five personality traits and life satisfaction, we conducted a simple linear regression model for life satisfaction, wherein factors of the Big Five personality were independent variables, and gender and age were control variables (see **Table 2**). Using the “enter” method of regression analysis, results indicated that, (a) after controlling for all other personality traits, extraversion, agreeableness, and conscientiousness significantly positively correlated with life satisfaction, respectively; (b) after controlling for all other personality traits, neuroticism significantly negatively correlated with life satisfaction; whereas (c) after controlling for all other personality traits, openness to new experience showed no significant association with life satisfaction.

**Table 2:** Regression analysis for effects of the Big Five factors on life satisfaction (*N* = 2,357).

	Variables	β	*F*	*R^2^*	Δ*R^2^*
Step 1			14.01***	0.01	
	Gender	0.02			
	Age	0.11***			
Step 2			192.32***	0.36	0.35
	Gender				
	Age	0.11***			
	E	0.27***			
	A	0.12***			
	O	0.00			
	C	0.22***			
	N	-0.27***			

^∗^*p< 0.05; ^∗∗^*p* < 0.01; aaa*p* < 0.001. E = Extraversion, A = Agreeableness, O = Openness to New Experience, C = Conscientiousness, N = Neuroticism*.

#### The Associations between the Big Five Personality Traits and Life Satisfaction: The Mediating Effect of Coping Style

**FIGURE 2:**
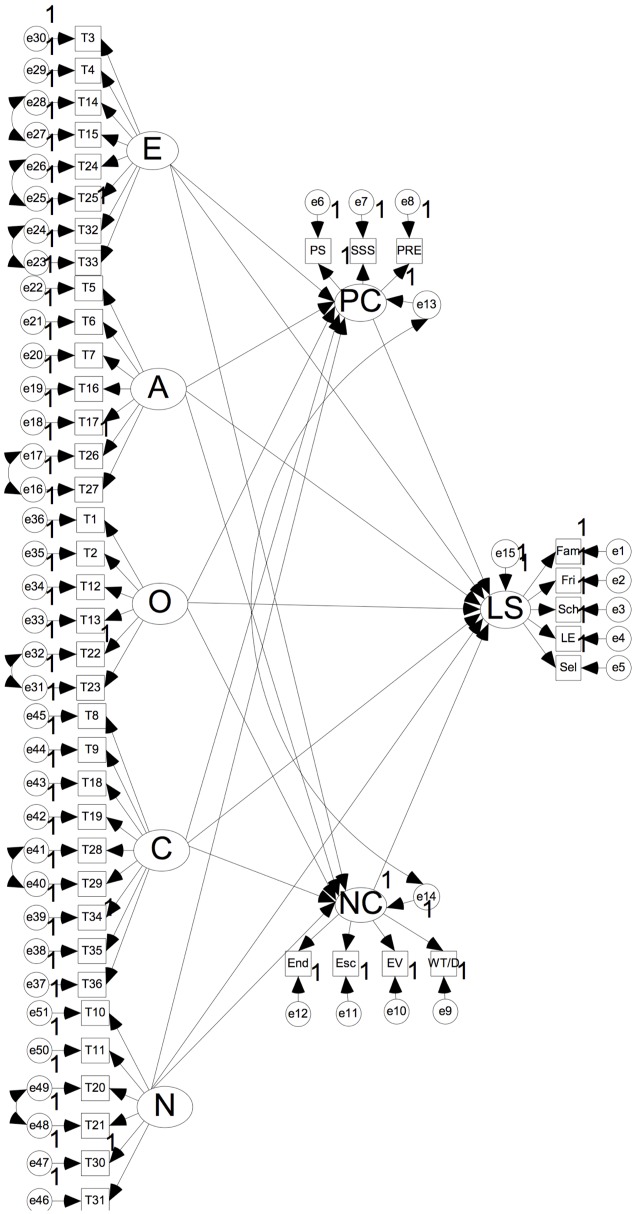
The mediation model of the Big Five personality traits on life satisfaction. Control variables were included in the model but not presented for simplicity. E = Extraversion, A = Agreeableness, 0 = Openness to New Experience, C = Conscientiousness, N = Neuroticism, PC = Positive Coping, PS = Problem Solving, SSS = Seeking Social Support, PRE = Positively Rationalized Explanation, NC = Negative Coping, End = Enduring, Esc = Escape, EV = Emotion Venting, WT/D = Wishful Thinking/Denial, LS = Life Satisfaction, Fam = Family Satisfaction, Fri = Friend Satisfaction, Sch = School Satisfaction, LE = Living Environment Satisfaction, Sel = Self Satisfaction.

Structural equation model (Joreskog and Sorbom, 1979) was performed by AMOS 17.0 to test our hypothesized mediation model, in which the factors of the Big Five personality were independent variables, gender and age were control variables, and the two categories of coping style were the mediating factors. Following the modification indices, the results of the modified SEM (see **Figure 2** and **Table 3**) indicated an acceptable fit: χ^2^ = 9997.01, *df* = 1124, χ^2^/*df* = 8.89, *p* < 0.001; RMSEA = 0.05; SRMR = 0.05; CFI = 0.89; NFI = 0.89; GFI = 0.90.

**Table 3:** Bootstrap analysis summary showing the indirect effects of the Big Five personality traits on life satisfaction via coping style (*N* = 2,357).

Independent variables (IV)	Mediator variable (MV)	Dependent variables (DV)	*a* Path coefficient (IV-MV)	*b* Path coefficient (MV-DV)	Indirect effects and 95%CI [lower, upper]	*c’* Path coefficient (direct effect)
E	PC	LS	0.04**	0.25***	0.01 [0.004,0.02]	0.31***
E	NC	LS	0.24***	-0.03*	–0.01 [–0.03, –0.001]	
A	PC	LS	0.14**	0.25***	0.04 [0.03,0.06]	0.08**
A	NC	LS	0.09	-0.03*	–0.003 [–0.004,0.002]	
O	PC	LS	0.17***	0.25***	0.04 [0.02,0.13]	-0.05
O	NC	LS	0.03*	-0.03*	–0.001 [–0.03, –0.001]	
C	PC	LS	0.42***	0.25***	0.11 [0.08,0.13]	0.21***
C	NC	LS	-0.25***	-0.03*	0.01 [0.003,0.02]	
N	PC	LS	-0.11***	0.25***	–0.03 [–0.05, –0.02]	-0.26***
N	NC	LS	0.62***	-0.03*	–0.02 [–0.04, –0.01]	

^∗^*p< 0.05; ^∗∗^*p* < 0.01; aaa*p* < 0.001. E = Extraversion, A = Agreeableness, O = Openness to New Experience, C = Conscientiousness, N = Neuroticism, PC = Positive Coping, NC = Negative Coping, LS = Life Satisfaction. These values are based on standardized path coefficients. Controlling for the effect of gender and age on all the observed variables*.

According to **Table 3**, the associations between most factors of the Big Five personality traits and life satisfaction were mediated by coping style. To be specific, the relations between three traits (extraversion, conscientiousness, and neuroticism) of the Big Five personality and life satisfaction were partially mediated by both PC and NC; meanwhile, the association between openness to new experiences and life satisfaction was fully mediated by both PC and NC. The correlation between agreeableness and life satisfaction was partially mediated by PC only.

To test the significance of mediation effects, we used a joint significance test and bootstrap examination method (with 1000 bootstrap samples). If the CIs did not include zero (*p* < 0.05), we concluded that the mediating effects were statistically significant (Preacher and Hayes, 2008). The results of bootstrap analysis revealed that the indirect effect of each personality trait of the Big Five on life satisfaction via PC was significantly different from zero (see **Table 3**). Meanwhile, the indirect effect of each personality trait (except agreeableness) of the Big Five on life satisfaction via NC was significantly different from zero (see **Table 3**).

In summary, the results of this study demonstrated that (a) four factors of the Big Five personality traits, except openness to new experiences, had significant associations with life satisfaction among adolescents. Specifically, extraversion, along with agreeableness and conscientiousness, were positively correlated with life satisfaction, while neuroticism was negatively correlated with life satisfaction; (b) both positive and NC style played a mediating role between four personality traits (except agreeableness) of the Big Five and adolescents’ life satisfaction, and the path from agreeableness to life satisfaction was mediated by PC only.

## Discussion

This study showed that extraversion had a significantly positive association while neuroticism had a significantly negative association with adolescents’ life satisfaction, which was consistent with results of previous research (Heaven, 1989; Huebner, 1991; Fogle et al., 2002; McKnight et al., 2002; Rigby and Huebner, 2005; Ho et al., 2008). Furthermore, this study revealed that the associations between the Big Five personality traits and adolescents’ life satisfaction were different, which confirmed the findings in the literature (Lounsbury et al., 2005; Wang, 2013; Suldo et al., 2015). The above results supported H1 of this study. This study also demonstrated full or partial mediation effects of coping style on the relations between the Big Five personality traits and life satisfaction, which verified H2 of this study. Integrating all these findings, we could provide a reasonable explanation for why the Big Five traits differed in their associations with life satisfaction in the literature, theoretical implications for future research on the relations between personality and life satisfaction, and practical implications for the improvement of adolescents’ life satisfaction.

### The Associations between the Big Five Personality Traits and Life Satisfaction

According to the descriptive statistics and correlation results of this study, extraversion, agreeableness, openness to new experiences, and conscientiousness positively correlated with life satisfaction, while neuroticism negatively correlated with life satisfaction among adolescents, which were consistent with the conclusions of Tian and Zheng (2007).

To further analyze the associations between the Big Five personality traits and adolescents’ life satisfaction by regression model, we found that, after controlling for all other personality traits, extraversion was positively associated with life satisfaction and neuroticism was negatively associated with life satisfaction, which concurred with previous findings (Fogle et al., 2002; McKnight et al., 2002; Rigby and Huebner, 2005; Ho et al., 2008; Wang, 2013). In addition, our results reflected that, after controlling for all other personality traits, agreeableness as well as conscientiousness was positively associated with adolescents’ life satisfaction, respectively, which were consistently verified by previous studies (e.g., Lounsbury et al., 2005). The results indicated that adolescents with higher levels of extraversion, agreeableness, and conscientiousness showed more satisfaction with their lives than adolescents with lower levels of extraversion, agreeableness and conscientiousness (Lounsbury et al., 2005), while adolescents with higher levels of neuroticism felt less happy and less content with their lives than adolescents with lower levels of neuroticism (Ho et al., 2008; Garcia, 2011).

Even though results of correlation analysis indicated that openness to new experiences was significantly correlated with life satisfaction, openness to new experiences did not explain unique variance in adolescents’ life satisfaction in the regression model of the present study, after controlling for all other personality traits, which was also reported by previous researchers (Lounsbury et al., 2005; Garcia, 2011). In other words, the association between openness to new experiences and life satisfaction was reduced to non-significant finding once the other personality traits were statistically controlled in the regression analysis, suggesting that this association might be due to some third personality trait. However, the findings of our mediation model declared that the association between openness to new experiences and adolescents’ life satisfaction was fully mediated by both PC and NC simultaneously. This is of interest to future researchers concerning the relations between each of the Big Five traits and coping style, and the relations between personality traits and life satisfaction. The differentiated associations between the Big Five traits and life satisfaction in previous studies might be due to the fact that some mediation factors that were disregarded (Joshanloo and Afshari, 2011; Van Dijk et al., 2012), such as coping style, partially or fully mediated these associations (McCrae and Costa, 1986). Because coping style was not measured in some previous studies, some traits of the Big Five might show no association with adolescents’ life satisfaction.

### The Mediating Effect of Coping Style

The results of the present study indicated that the links between most traits of the Big Five and life satisfaction were mediated by coping style. Personality correlates with quality of life because it influences the ways individuals cope with stressful situations (Vollrath, 2001; Wrosch and Scheier, 2003; Vollrath and Landolt, 2005; Aarstad et al., 2008). Adolescents have an urgent need to construct relationships with peers and independently explore the outside world (Caspi, 1998). Meanwhile, they have to be responsible for their academic work and to act as appropriate role models in society (Compas et al., 2001). There are a number of stressors that adolescents have to cope with, and the choices of coping strategies further influence adolescents’ life satisfaction.

Based on our findings, the relation between extraversion and adolescents’ life satisfaction was partially mediated by both PC and NC, wherein extraversion was positively associated with PC and also was positively associated with NC; and in turn PC was positively associated with life satisfaction, while NC was negatively associated with life satisfaction. The findings revealed that adolescents high in extraversion frequently used both PC and NC. Choosing PC led to high life satisfaction whereas choosing NC led to low life satisfaction. Here, the finding that extraversion was positively associated with PC was consistent with prior research (e.g., Li and Zhang, 2004; Connor-Smith and Flachsbart, 2007). It might be contradictory to one’s intuitive thought that extraversion was positively associated with NC. However, it has been verified by many previous studies (Li et al., 2000; Li and Zhang, 2004; Zhou et al., 2017), which reported that extraversion was positively associated with emotion-focused coping. Extraversion has dual nature (Zhou et al., 2017). In terms of the essential component of extraversion, individuals with high extraversion often develop friendly relationships with others and have solid social support (Zhou et al., 2017), so they could frequently choose PC. However, extraverted individuals also have distinct characteristics of impulsiveness ((Eysenck and Eysenck, 1963). They tend to seek new stimulation (Eysenck, 1967) and are sensitive to reward (Carver and Connor-Smith, 2010). These factors were assumed to be highly associated with emotion-focused coping (Xue and Liang, 2012). In addition, some researchers found that extraversion was associated with negative emotions, which in turn influenced people’s subjective well-being (Costa and McCrae, 1980; McCrae and Costa, 1991; Masthoff et al., 2007). Other reasons need to be explored in future studies.

Second, the association between neuroticism and adolescents’ life satisfaction was partially mediated by both PC and NC, wherein neuroticism was negatively associated with PC and in turn PC positively associated with life satisfaction; and neuroticism was positively associated with NC and in turn NC was negatively associated with life satisfaction. This finding concurred with previous literature (Aarstad et al., 2008; Vollrath, 2001). Because adolescents encounter a number of stressors coming from school, friends, and family (Compas et al., 2001; Liu et al., 2016), adolescents’ emotions may be unstable (Yue et al., 2006). Therefore, adolescents with high neuroticism may feel much more anxious and impulsive, and they may choose NC strategies more frequently if they do not effectively control their emotions (Cook and Heppner, 1997), such as escaping challengeable situations (Eby et al., 2005), denying reality (Connor-Smith and Flachsbart, 2007; Fickova, 2009), and so on, which contributes to adolescents feeling unhappy about their lives.

Next, the relation between agreeableness and adolescents’ life satisfaction was partially mediated by PC only, wherein agreeableness was positively associated with PC, and in turn PC was positively associated with life satisfaction. Agreeableness negatively correlated with NC but did not show significant association with NC. These results suggested that adolescents high in agreeableness chose NC less frequently; adolescents with high scores on agreeableness use PC much more frequently, and then the outcome of PC was feeling happy about life. It seemed that adolescents with high agreeableness were friendly and peaceful (Caspi, 1998), and they could seek social supports from their social networks, which was helpful for them to actively cope with stress (Baltes et al., 2011) and enhance their evaluation of life.

In addition, this study showed that the association between openness to new experiences and adolescents’ life satisfaction was fully mediated by both PC and NC simultaneously, wherein openness to new experiences was positively associated with PC and was positively associated with NC, and in turn PC was positively associated with life satisfaction, while NC was negatively associated with life satisfaction. High openness to new experiences allowed adolescents to show a wide range of interests for the outside (Zhou et al., 2017) and to restructure cognition for stressful events (Caspi, 1998), which may be helpful for adolescents to optimistically cope with challenges. However, adolescents with high openness to new experiences might rely on passive imagination of reality to escape stressors, or act in non-conforming ways (Masthoff et al., 2007; Van De Ven and Engels, 2011), which may promote adolescents to choose NC, such as wishful thinking (Carver and Connor-Smith, 2010). To explore the potential reasons why openness to new experiences was positively associated with both PC and NC should be the focus of future studies.

Furthermore, the path from conscientiousness to adolescents’ life satisfaction was partially mediated by both PC and NC, wherein conscientiousness was positively associated with PC, and in turn PC was positively associated with life satisfaction; conscientiousness was negatively associated with NC, and in turn NC was negatively associated with life satisfaction. Conscientiousness is characterized by responsibility and intentionality (Caspi, 1998). Adolescents with high conscientiousness may have had a stronger sense of responsibility and self-discipline, so they could make a plan and be persistent in problem solving (Connor-Smith and Flachsbart, 2007), and these PC strategies were beneficial for problem solving, which eventually led to high levels of life satisfaction.

In short, the mediating effect of coping style explained why the Big Five traits differed in their associations with life satisfaction, which was also found in previous literature. It was plausible to infer that the reason why some traits were still found to have significant associations with adolescents’ life satisfaction was because some ignored variables (such as the coping style in this study) might have been left unexamined, which just partially mediated the association between those traits and life satisfaction. These kinds of potential mediating variables were not studied (such as Lounsbury et al., 2005; Wang, 2013; Suldo et al., 2015). It was possible that some other potential mediating factors, such as self-esteem (Joshanloo and Afshari, 2011; Tan et al., 2016), approach behavioral motivation, and avoidance behavioral motivation (Carver and Scheier, 1998; Van Dijk et al., 2012), could also partially mediate the associations between personality traits and adolescents’ life satisfaction. However, personality traits differed in their associations with life satisfaction (such as Garcia, 2011), especially some personality traits showed no significant associations with adolescents’ life satisfaction, which was the same as the finding that openness to new experiences showed no significant association with adolescents’ life satisfaction in the present study. One of the reasonable explanations was that coping style fully mediated the paths from personality traits to adolescents’ life satisfaction, which made the associations between some personality traits and adolescents’ life satisfaction to be non-significant.

### Implications for Educational Practice and Future Research

This study confirmed the associations between some of the Big Five personality traits and adolescents’ life satisfaction, which had been demonstrated in preceding literature, and also verified the mediating effect of coping style on the relations between the Big Five personality traits and life satisfaction. These findings were helpful to explain why the Big Five traits differed in their associations with life satisfaction. In other words, exploring the mediation mechanism from personality to life satisfaction can give us further understanding about how personality traits were associated with life satisfaction. In addition to coping style addressed in this study, some other variables such as self-esteem, approach behavioral motivation, and avoidance behavioral motivation probably also have such mediating effects. Given the fact that these other possible mediating factors had high correlations with personality and coping style, examining the chain mediation effects among these variables would be meaningful to extend our understanding of the relations between personality and life satisfaction in the future.

This study showed that the associations between personality traits and adolescents’ life satisfaction were fully or partially mediated by coping style, which suggested that both personality and coping style should be taken into account in order to improve adolescents’ life satisfaction. Providing corresponding coping style intervention such as problem solving, seeking social support, or positive rationalization to adolescents in terms of their personality traits would be helpful to facilitate overcoming their difficulties with school, friends, and family, and then increasing their life satisfaction during the critical periods of their life.

Third, the particular characteristics of our participants inspired future research. The relations among personality traits, coping style and life satisfaction with different age groups (e.g., adolescents, adults) or with adolescents from different cultural background should be explored in the future research. Life satisfaction of adults has been studied extensively (e.g., Zhang and He, 2010), whereas little attention has been paid to the youth (Proctor et al., 2009; Weber and Huebner, 2015). Differences between different age groups (undergraduate samples and adult samples) were found in the associations of the Big Five personality traits with life satisfaction (Acevedo, 2010). Given that life events of different age groups (e.g., adolescents, adults) are different (e.g., study, work), individuals would have variable evaluations for their lives. To further examine the association among personality, coping style and life satisfaction between adolescents and other different age groups would benefit studies regarding the predictors of life satisfaction through a developmental perspective. On the other hand, cultural contexts (e.g., birth order) were correlated with personality and life satisfaction (Sulloway, 1996, 2001; Shao et al., 2013). To verify the external validity of this study in other countries would advance our understanding of cross-cultural differences in the relations between personality and life satisfaction. Furthermore, it would be our future interests to assess the correlations among personality, coping style and life satisfaction in a bidirectional way.

### Limitations and Further Directions

Though the findings of this study are promising, there are several limitations. First, variables were assessed by questionnaires at one moment, so it was impossible to explore dynamic relations among the Big Five personality traits, coping style, and life satisfaction over a longer period. Surveying the continuous process of these variables by longitudinal studies is warranted in the future. Second, social desirability is an inevitable problem when using questionnaires to collect data. Adding some objective behaviors as indicators could be considered in future studies. Third, due to adolescents’ life satisfaction including evaluation of different aspects of life, it is better to analyze the mediation effect of coping style between personality traits and satisfaction of different aspects of life, rather than considering life satisfaction as a general factor processed in the present study.

## Author Contributions

LX designed the study and wrote the manuscript. R-DL provided instruction and advise for the study. YD revised the grammar of the manuscript and edited its format. XM conducted the survey and trained research assistants. JW was in charge of data analysis. YL was responsible for checking the results.

## Conflict of Interest Statement

The authors declare that the research was conducted in the absence of any commercial or financial relationships that could be construed as a potential conflict of interest.
