# Supplementary material for: The Mediation Effect of Coping Style on the Relations between Personality and Life Satisfaction in Chinese Adolescents
Source: Front Psychol. 2017 Jun 29;8:1076. doi: 10.3389/fpsyg.2017.01076 (PMC5489605; doi:10.3389/fpsyg.2017.01076)
Supplement: Supplementary file 1 [file Data_Sheet_1.PDF]

Appendix

The following content is related to you. Please answer these questionnaires truly with your own opinion. Please mark circles ("O") on the numbers that you choose.

For example:

One item was written as "I always talk with my parents". Its answers include five options: strongly disagree, largely disagree, slightly agree, largely agree and strongly agree. The numbers for these five options are 1, 2, 3, 4 and 5. Specifically, "strongly disagree" means that the description of this item is totally unsuitable for you anytime; "largely disagree" means that the description of this item is unsuitable for you most of the time; "slightly agree" means that the description of this item is suitable for you sometime but unsuitable for you in some other time; "largely agree" means that the description of this item is suitable for you most of the time; "strongly agree" means that the description of this item is totally suitable for you anytime. For example, if you think the description of this item is unsuitable for you most of the time, you should chose the "largely disagree" option and mark "O" on the number 2.

Please carefully answer the following items now.

Adolescents' Five-Factor Personality Questionnaire (Revised Version, PE)

Everyone shows his/her own characteristics by behaviors, and these characteristics make everyone to be unique. There are no good characteristics or bad characteristics. Some familiar behaviors are listed below and please choose an answer for each item according to your actual situation. Please mark "O" on the numbers that you select.

Items		Strongly Disagree	Largely Disagree	Slightly Agree	Largely Agree	Strongly Agree
PE01	My understanding ability is good, so I learn new knowledge quickly.	1	2	3	4	5

PE02	My observation ability is sensitive, so I can observe some details that others can not find.	1	2	3	4	5
PE03	I like playing with classmates.	1	2	3	4	5
PE04	I can join a new team quickly.	1	2	3	4	5
PE05	I can understand others and consider others' feelings.	1	2	3	4	5
PE06	I help others as far as possible.	1	2	3	4	5
PE07	Once I promise, I will try my best to finish the task.	1	2	3	4	5
PE08	I work conscientiously, and I carefully check after I finish a work to avoid mistakes.	1	2	3	4	5
PE09	With or without others' presence, I can restrain my own behaviors.	1	2	3	4	5
PE10	I often feel unhappy, and not happy for a few days.	1	2	3	4	5
PE11	I often worry about bad things happening.	1	2	3	4	5
PE12	In a play or other activities, I often think of the novel ideas.	1	2	3	4	5
PE13	I have my own unique opinions for a lot of things.	1	2	3	4	5
PE14	I am good at communication and like to talk with others.	1	2	3	4	5
PE15	I am very enthusiastic, and I often take the initiative to make new friends.	1	2	3	4	5
PE16	I am warm and friendly to people.	1	2	3	4	5
PE17	I treat people sincerely.	1	2	3	4	5
PE18	I always study hard.	1	2	3	4	5

PE19	I strive to do things perfectly and accurately.	1	2	3	4	5
PE20	I often get angry because of a small thing.	1	2	3	4	5
PE21	I'm easy to be emotional.	1	2	3	4	5
PE22	I have a rich imagination.	1	2	3	4	5
PE23	I like the freedom to fantasize.	1	2	3	4	5
PE24	I am an optimistic and cheerful person.	1	2	3	4	5
PE25	I love to laugh and also like to joke with others.	1	2	3	4	5
PE26	I never force or control other people.	1	2	3	4	5
PE27	I don't impose my ideas on others.	1	2	3	4	5
PE28	I pay attention to keep clean and tidy, and my items are organized.	1	2	3	4	5
PE29	I can do things orderly, and finish work step by step as planned.	1	2	3	4	5
PE30	I often feel helpless. I think no one can help me.	1	2	3	4	5
PE31	Compared with the people around, I often feel inferior to others.	1	2	3	4	5
PE32	People usually obey my arrangement in events.	1	2	3	4	5
PE33	I often act as a leader to organize some extracurricular activities.	1	2	3	4	5
PE34	I am a trustworthy person.	1	2	3	4	5
PE35	As long as I decided to do something, I don't give up easily.	1	2	3	4	5
PE36	I try my best to complete the tasks that teachers or parents	1	2	3	4	5

	give to me.					

Coping Style Scale for Middle School Students (CS)

Everyone encounters many challenges or confusing things in daily life, but everyone has his own method to cope with these troubles. Here are a few coping methods that people usually utilize when they are in trouble. Please imagine how you will cope when you meet difficulties or troubles. There are no good coping methods or bad coping methods. Please choose an answer for each item according to your actual situation and mark "O" on the numbers that you select.

Items		Never Use	Seldom Use	Sometime Use	Always Use
CS01	To use my own or others' experience to cope with difficulties.	1	2	3	4
CS02	To consult others who have similar experiences.	1	2	3	4
CS03	To consider some good aspects from the bad things that have happened.	1	2	3	4
CS04	I think that "To take a step back to gain more." when I encounter difficulties.	1	2	3	4
CS05	I have a fantasy that I can use superman's abilities to overcome difficulties.	1	2	3	4
CS06	I try my best to change the situation to make things to become better.	1	2	3	4
CS07	Trying to learn "what should I do" from others' advice.	1	2	3	4
CS08	Try to change my perspective and I can see the positive side of the setback.	1	2	3	4
CS09	Encountering a setback, I give up on efforts to get what I want.	1	2	3	4
CS10	I love doing some unrealistic things to eliminate worry.	1	2	3	4
CS11	Thinking seriously about what is the best way to solve the problem.	1	2	3	4
CS12	Seeking support from classmates, family members or relatives to overcome the difficulties.	1	2	3	4
CS13	I learned some useful things from hard experience.	1	2	3	4
CS14	Admitting that I can't deal with the	1	2	3	4

	problem at hand, and stop attempting.				
CS15	I often think that "this is not true" in the face of difficulties.	1	2	3	4
CS16	Trying my best to find a solution to the problem.	1	2	3	4
CS17	Telling confusing things to others.	1	2	3	4
CS18	I regard difficulties and setbacks as a part of the life experience.	1	2	3	4
CS19	I often tell myself "to be patient" when facing a setback.	1	2	3	4
CS20	Venting bad emotions and becoming confused when I encounter troubles.	1	2	3	4
CS21	I often hope that the problem has been solved when I wake up.	1	2	3	4
CS22	Making plans to solve the problem, and carrying out the plan step by step.	1	2	3	4
CS23	I want to get emotional support from family, relatives, or friends.	1	2	3	4
CS24	I think life is suffering.	1	2	3	4
CS25	I bury the unpleasant things in my heart.	1	2	3	4
CS26	Losing my temper because of difficulties.	1	2	3	4
CS27	Refusing to believe that bad things have happened.	1	2	3	4
CS28	Learning lessons from the past failures and using experiences to solve the difficulties in the future.	1	2	3	4
CS29	Expecting to gain sympathy and understanding from others.	1	2	3	4
CS30	For some unpleasant things what I can do is only enduring, because my ability is limited.	1	2	3	4
CS31	Encountering troubles, I give up or reduce my goals.	1	2	3	4
CS32	If I can not solve problems, I will be upset and vent bad emotions to families and friends.	1	2	3	4
CS33	Doing something to solve the problem slowly.	1	2	3	4
CS34	Discussing with classmates, friends, or families to think of solutions for the problem.	1	2	3	4
CS35	To take waiting or unconcerned attitude for troubles.	1	2	3	4
CS36	Finding some ways to vent bad emotions.	1	2	3	4

Multidimensional Students' Life Satisfaction Scale in Chinese Version (LS)

The following items are related to you. Please answer these questionnaires truly with your own opinion. Please mark circles ("O") on the numbers that you choose.

Items		Strongly Disagree	Largely Disagree	Largely Agree	Strongly Agree
LS01	There is a good relationship in my family.	1	2	3	4
LS02	I like to stay together with my family.	1	2	3	4
LS03	School is an interesting place.	1	2	3	4
LS04	I like to go to school.	1	2	3	4
LS05	I learn a lot in school.	1	2	3	4
LS06	I often do some interesting things with my families.	1	2	3	4
LS07	I like the neighbors near my house.	1	2	3	4
LS08	I like the environment around my house.	1	2	3	4
LS09	Families are very fair to me.	1	2	3	4
LS10	My families talk with each other in a friendly way.	1	2	3	4
LS11	I am a good person.	1	2	3	4
LS12	Most people like me.	1	2	3	4
LS13	Being together with me is very interesting.	1	2	3	4
LS14	I feel very happy when staying with friends.	1	2	3	4
LS15	I like to stay in school.	1	2	3	4
LS16	I like to participate in school activities.	1	2	3	4
LS17	Our house is very comfortable to live in.	1	2	3	4
LS18	There are a lot of interesting things near my home.	1	2	3	4
LS19	I like the place where I live.	1	2	3	4
LS20	My friends are good to me.	1	2	3	4
LS21	If I need help, friends will help me.	1	2	3	4
LS22	My friends are very nice.	1	2	3	4
LS23	My friends are very friendly to me.	1	2	3	4
LS24	I feel I look good.	1	2	3	4
LS25	I like myself.	1	2	3	4
